# A Conserved Pattern of Primer-Dependent Transcription Initiation in *Escherichia coli* and *Vibrio cholerae* Revealed by 5′ RNA-seq

**DOI:** 10.1371/journal.pgen.1005348

**Published:** 2015-07-01

**Authors:** Sergey Y. Druzhinin, Ngat T. Tran, Kyle S. Skalenko, Seth R. Goldman, Jared G. Knoblauch, Simon L. Dove, Bryce E. Nickels

**Affiliations:** 1 Department of Genetics and Waksman Institute, Rutgers University, Piscataway, New Jersey, United States of America; 2 Division of Infectious Diseases, Boston Children's Hospital, Harvard Medical School, Boston, Massachusetts, United States of America; The University of Texas Health Science Center at Houston, UNITED STATES

## Abstract

Transcription initiation that involves the use of a 2- to ~4-nt oligoribonucleotide primer, “primer-dependent initiation,” (PDI) has been shown to be widely prevalent at promoters of genes expressed during the stationary phase of growth in *Escherichia coli*. However, the extent to which PDI impacts *E*. *coli* physiology, and the extent to which PDI occurs in other bacteria is not known. Here we establish a physiological role for PDI in *E*. *coli* as a regulatory mechanism that modulates biofilm formation. We further demonstrate using high-throughput sequencing of RNA 5′ ends (5′ RNA-seq) that PDI occurs in the pathogenic bacterium *Vibrio cholerae*. A comparative global analysis of PDI in *V*. *cholerae* and *E*. *coli* reveals that the pattern of PDI is strikingly similar in the two organisms. In particular, PDI is detected in stationary phase, is not detected in exponential phase, and is preferentially apparent at promoters carrying the sequence T_−1_A_+1_ or G_−1_G_+1_ (where position +1 corresponds to the position of *de novo* initiation). Our findings demonstrate a physiological role for PDI and suggest PDI may be widespread among Gammaproteobacteria. We propose that PDI in both *E*. *coli* and *V*. *cholerae* occurs though a growth phase-dependent process that leads to the preferential generation of the linear dinucleotides 5´-UA-3´ and 5´-GG-3´.

## Author Summary

Primer-dependent transcription initiation, PDI, refers to an alternative mechanism of transcription initiation whereby the first phosphodiester bond within the nascent RNA is formed between a 2- to ~4-nt RNA primer and an incoming nucleoside triphosphate. Although PDI has been shown to occur in *E*. *coli*, the impact of PDI on *E*. *coli* physiology, and the extent to which PDI occurs in other bacteria is unknown. Here we establish that PDI modulates the ability of *E*. *coli* to form biofilms, a surface attached community of bacteria encased in a polymeric matrix. We further describe a significantly improved RNA-seq based method for the detection of PDI in cells. Using this method we document the occurrence of PDI in the pathogenic bacterium *Vibrio cholerae*. We further show that the pattern of PDI in *V*. *cholerae* is identical to that observed in *E*. *coli*, suggesting that PDI in these two organisms may occur through a conserved process that produces identical populations of 2- to ~4-nt RNA primers. Our findings suggest PDI may be widespread in Gammaproteobacteria.

## Introduction

Transcription in all cells is carried out by multi-subunit RNA polymerases (RNAPs) that are conserved in sequence, structure, and function from bacteria to humans. The first step in transcription, initiation, consists of a number of discrete steps that culminate in the RNAP-mediated catalysis of the first phosphodiester bond formed within the nascent RNA [1, 2]. The first phosphodiester bond within the nascent RNA can be formed between two nucleoside triphosphate (NTP) substrates, “*de novo* initiation,” or between a 2- to ~4-nt oligoribonucleotide primer and an incoming NTP, “primer-dependent initiation,” PDI. Although PDI had been long known to occur during transcription reactions performed *in vitro* (reviewed in [3]), PDI has only recently been shown to occur during the stationary phase of growth in *Escherichia coli* [4, 5]. In addition, the extent of PDI relative to *de novo* initiation at a given promoter *in vivo* can influence the overall abundance of transcripts produced from the promoter as well as the sequence and phosphorylation state of the 5′ ends of transcripts produced from the promoter [3–6].

**Fig 1:**
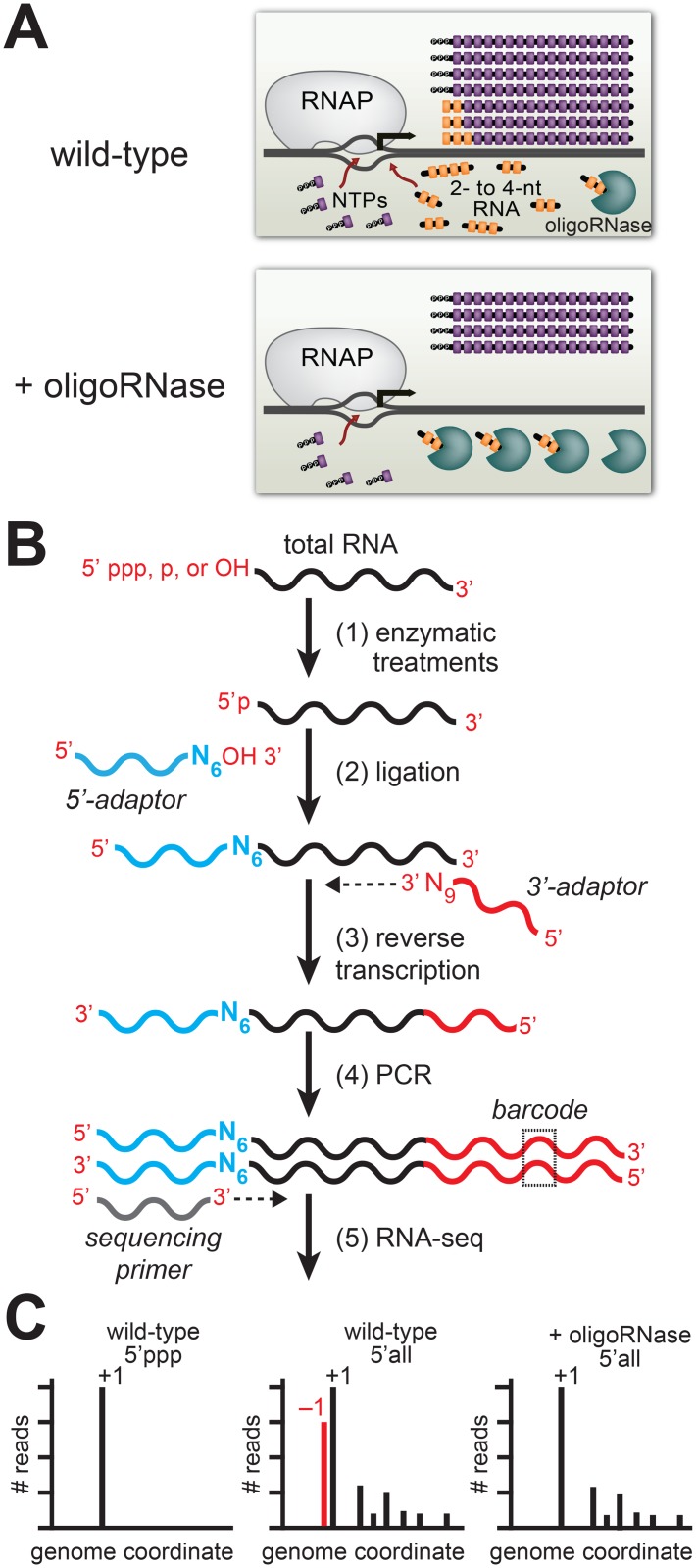
Detection of PDI in bacteria by ectopic expression of an oligoRNase coupled with 5′ RNA-seq. A. Inhibition of PDI through ectopic expression of an oligoRNase. Depicted is the extent of *de novo* initiation and PDI from a representative promoter in wild-type cells (top) and cells in which an oligoRNase is ectopically produced (bottom). 2- to 4-nt oligoribonucleotides, are depicted in yellow while NTPs are shown in purple. In the example shown here, PDI leads to generation of full-length transcripts that do not carry a 5′ triphosphate and are one base longer than the products of *de novo* initiation. B. Steps in 5′ RNA-seq [16]. Step 1: selective enzymatic treatments of total RNA that allow cDNA libraries to be constructed from RNAs on the basis of the phosphorylation state of the 5′ end. Step 2: ligation of 5′ adaptor (in blue) carrying six random bases (N_6_) at the 3′ end. Step 3: Reverse transcription using a primer with sequence of the Illumina 3′ adaptor on the 5′ end and nine random bases at the 3′ end (N_9_). Step 4: PCR step includes a primer that introduces a barcode (dashed box) enabling several libraries to be analyzed, in parallel, on the Illumina HiSeq. C. Histograms generated from 5′ RNA-seq. Analysis of transcripts with a 5′ triphosphate (5′ ppp) is used to identify primary start sites (+1). Comparison of results obtained from wild-type cells or cells in which an oligoRNase is ectopically expressed using the analysis of the 5′ ends of all transcripts (i.e. those carrying a 5′-triphosphate, 5′-monophosphate, or 5′-hydroxyl) identifies transcripts generated by PDI that emanate from position −1 (red).

To detect PDI *in vivo* we developed the experimental pipeline shown in Fig 1 [5] that is based on two experimental considerations. First, studies of RNA metabolism in bacteria indicate that 2- to ~4-nt oligoribonucleotides (species that are sometimes referred to as “nanoRNAs”) are degraded in cells by specialized ribonucleases termed “oligoribonucleases (oligoRNase)” or “nanoRNases” [7–9]. Thus, by increasing the concentration of an oligoRNase *in vivo* we decrease the concentrations of 2- to ~4-nt oligoribonucleotides *in vivo* (Fig 1A). Second, *in vitro* analyses indicate that 2- to 4-nt oligoribonucleotides effectively compete with NTPs for use as transcription primers provided the 5′ end of the RNA is complementary to sequences between positions −3 and +1 (where +1 is the position of *de novo* initiation) and the 3′ end is complementary to positions +1, +2 or +3 [6, 10–15]. Thus, PDI with 2- to 4-nt oligoribonucleotides *in vitro* leads to the generation of transcripts emanating from template position +1 or template positions upstream of +1 (−3, −2, or −1). To unambiguously distinguish transcripts generated by PDI from those generated by *de novo* initiation we use high-throughput sequencing of RNA 5′ ends (5′ RNA-seq) [16] to first, identify the primary *de novo* start sites associated with promoters genome-wide, and second, identify transcripts that emanate from template positions upstream of these primary *de novo* start sites whose abundance decreases upon ectopic expression of an oligoRNase (Fig 1B and 1C).

Our 5′ RNA-seq procedure facilitates the analysis of both the sequence and phosphorylation state of the portion of the transcriptome comprising the 5′ ends of RNAs. Because transcripts generated by *de novo* initiation carry a 5′ triphosphate the analysis of transcripts that carry a triphosphate group can be used to identify primary *de novo* start sites (designated position +1), each with its associated “start site region” (i.e. positions −3 to +4) (Fig 1C, left histogram). Next, we use the analysis of the 5′ ends of all transcripts (i.e. those carrying a 5′-triphosphate, 5′-monophosphate, or 5′-hydroxyl) to determine the effect of ectopic expression of an oligoRNase on the fraction of transcripts initiated from positions upstream of +1 within each start site region (Fig 1C, middle and right histograms). The inclusion of 5′-monophosphate- and 5′-hydroxyl-containing transcripts in the analysis allows us to identify PDI events that involve a primer carrying either a 5′-monophosphate or a 5′-hydroxyl. Using this experimental pipeline we established that PDI occurs in *E*. *coli* and is growth phase-dependent [4, 5]. Specifically, we found that PDI is detected during stationary phase but is not detected during exponential phase. In addition, we found that the growth phase-dependent PDI detected in *E*. *coli* leads to a significant increase in the stationary phase expression of at least two genes, *bhsA* and *tomB* [5].

Although PDI has been shown to occur in *E*. *coli*, the impact of PDI on *E*. *coli* physiology, and the extent to which PDI occurs in other bacteria is unknown. Thus, determining the full scope of PDI in *E*. *coli* and other bacteria, the mechanisms allowing or restricting PDI in *E*. *coli* and other bacteria, the specificity with which PDI is targeted to specific promoters in *E*. *coli* and other bacteria, and the role that PDI plays in cell growth in *E*. *coli* and other bacteria are significant open questions.

Here we present evidence that, in *E*. *coli*, the PDI-dependent increase in *bhsA* expression that occurs during stationary phase contributes to biofilm formation. Thus, our findings illuminate a previously undocumented physiological role for PDI in *E*. *coli* as a regulatory mechanism involved in biofilm growth. In addition, using a modified version of our experimental pipeline that employs a more sensitive 5′ RNA-seq protocol, we document the occurrence of PDI in the pathogenic Gammaproteobacteria *Vibrio cholerae*. We further find that the pattern of PDI observed in *V*. *cholerae* is strikingly similar to that observed in *E*. *coli*. Specifically, PDI in both *V*. *cholerae* and *E*. *coli* is detected during stationary phase, is not detected during exponential phase, and is preferentially targeted to promoters carrying start site regions with the sequence T_−1_A_+1_ or G_−1_G_+1_ (where position +1 corresponds to the position of *de novo* initiation). PDI from promoters carrying T_−1_A_+1_ and G_−1_G_+1_ start site regions produces full-length transcripts emanating from position −1 that carry a 5′ hydroxyl and begin with the sequence 5′-UA and 5′-GG, respectively. Thus, the primers used to generate these transcripts must themselves begin with the sequence 5′-UA and 5′-GG, respectively. We propose that a growth phase-dependent process that preferentially generates 5′-UA and 5′-GG oligoribonucleotides occurs in both *E*. *coli* and *V*. *cholerae*.

## Results

### PDI contributes to biofilm formation in *E*. *coli*

In prior work [5], we showed that PDI in *E*. *coli* leads to an increase in the stationary phase expression of *bhsA*, a gene that encodes a small outer membrane protein. In particular, we found that ~80% of transcripts emanating from a promoter associated with *bhsA* during stationary phase are produced as a consequence of PDI. Furthermore, ectopic expression of an oligoRNase reduces the expression of *bhsA* ~4-fold during the stationary phase of growth but does not alter *bhsA* expression during exponential phase.

**Fig 2:**
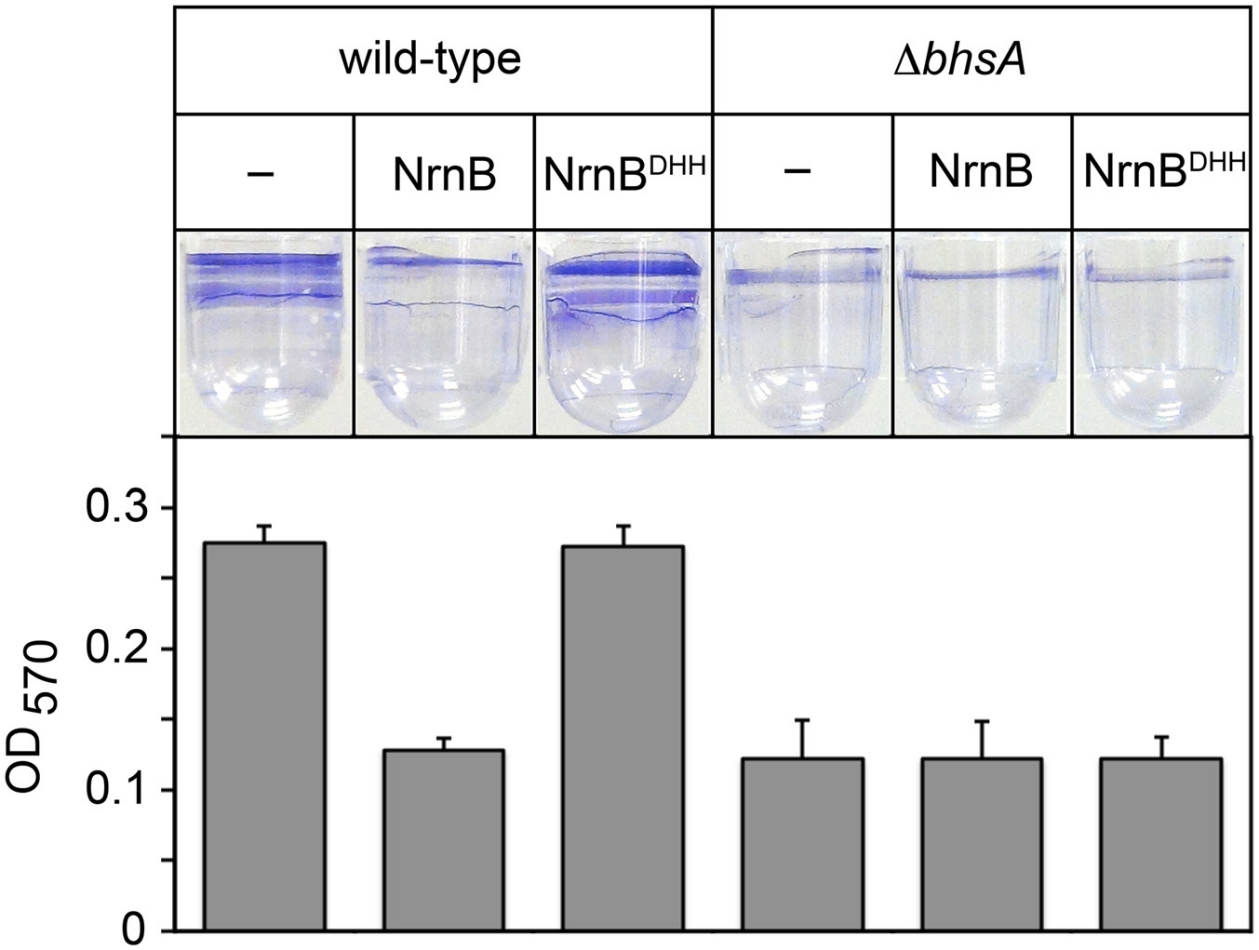
The PDI-dependent increase in *bhsA* expression contributes to biofilm formation in *E*. *coli*. Top shows images of representative wells containing biofilms stained with crystal violet dye. Graph shows averages +SEM of the amount of crystal violet staining observed from at least four independent measurements. Assays were done using wild-type MG1655 cells or MG1655 cells carrying a deletion of *bhsA* (*ΔbhsA*). Cells carried an empty plasmid (–) or a plasmid that directs the synthesis of NrnB or NrnB^DHH^.

Work from others has shown that *bhsA* influences the ability of *E*. *coli* cells to form biofilms [17], a mode of growth that results in the formation of a surface attached community of bacteria encased in a polymeric matrix. We therefore sought to address whether or not the changes in *bhsA* expression that occur as a consequence of PDI contribute to the ability of *E*. *coli* cells to form biofilms. To do this, we determined the effect of ectopic expression of a heterologous oligoRNase, *Bacillus subtilis* NrnB [9], or a catalytically inactive mutant, NrnB^DHH^ [9], on biofilm formation in wild-type *E*. *coli*, or a mutant strain in which *bhsA* has been deleted (*ΔbhsA*). We placed cell suspensions into wells of a microtiter plate, allowed biofilms to form by incubating for 32 hours at 25°C without agitation, and quantified the extent of biofilm formation using a standard crystal violet staining assay [18] (Fig 2). Ectopic expression of NrnB, but not NrnB^DHH^, caused a ~2-fold reduction in biofilm formation in wild-type *E*. *coli* (Fig 2). Furthermore, deletion of *bhsA* also caused a ~2-fold reduction in biofilm formation (Fig 2). However, in contrast to what we found in wild-type *E*. *coli*, ectopic expression of NrnB in *ΔbhsA* cells had no effect on the ability of these cells to form biofilms (Fig 2). Thus, the reduction in biofilm formation observed upon ectopic expression of NrnB in wild-type cells requires the presence of BhsA. We conclude that the increase in *bhsA* expression that occurs as a consequence of PDI contributes to biofilm formation in wild-type *E*. *coli*. These findings reveal a specific role for PDI as a regulatory mechanism that modulates biofilm formation in *E*. *coli*.

### Analysis of PDI in *Vibrio cholerae* by ectopic expression of an oligoRNase coupled with 5′ RNA-seq

Having established that PDI in *E*. *coli* can impact stationary phase gene expression [5] and cell physiology (Fig 2) we wished to investigate the occurrence of PDI in other bacteria. We therefore sought to define the extent of PDI in the pathogenic Gammaproteobacteria *V*. *cholerae* by employing the experimental pipeline used to detect PDI in *E*. *coli* (Fig 1). Thus we determined the effect of ectopically producing NrnB in *V*. *cholerae* on the distribution of transcription start sites as detected by 5′ RNA-seq.

For our analysis of PDI in *V*. *cholerae* we employed a revised 5′ RNA-seq protocol [16] that differed from that employed in our prior analysis of PDI in *E*. *coli* [5]. In particular, the 5′ and 3′ adaptors used in the revised 5′ RNA-seq protocol enable construction of cDNA compatible with analysis on the Illumina HiSeq system, whereas our prior *E*. *coli* analysis was done using 5′ and 3′ adaptors that enable construction of cDNA libraries compatible with the Applied Biosystems SOLiD system. In addition, the 5′ adaptor used in the revised protocol carries six randomized bases at the 3′ end (see Fig 1B), whereas our prior *E*. *coli* analysis was done using a 5′ adaptor that did not carry randomized bases at the 3′ end.

Having previously found that PDI in *E*. *coli* occurs during the stationary phase of growth we determined the effect of ectopically producing NrnB on the distribution of transcription start sites in *V*. *cholerae* during stationary phase. We identified several start site regions where the percentage of transcripts emanating from template positions upstream of the primary *de novo* start site was reduced by ectopic expression of NrnB (S1 and S2 Tables). Among these start site regions, we identified 10 where ectopic expression of NrnB reduced the percentage of transcripts emanating from upstream template positions by >25% (S1 Table).

**Fig 3:**
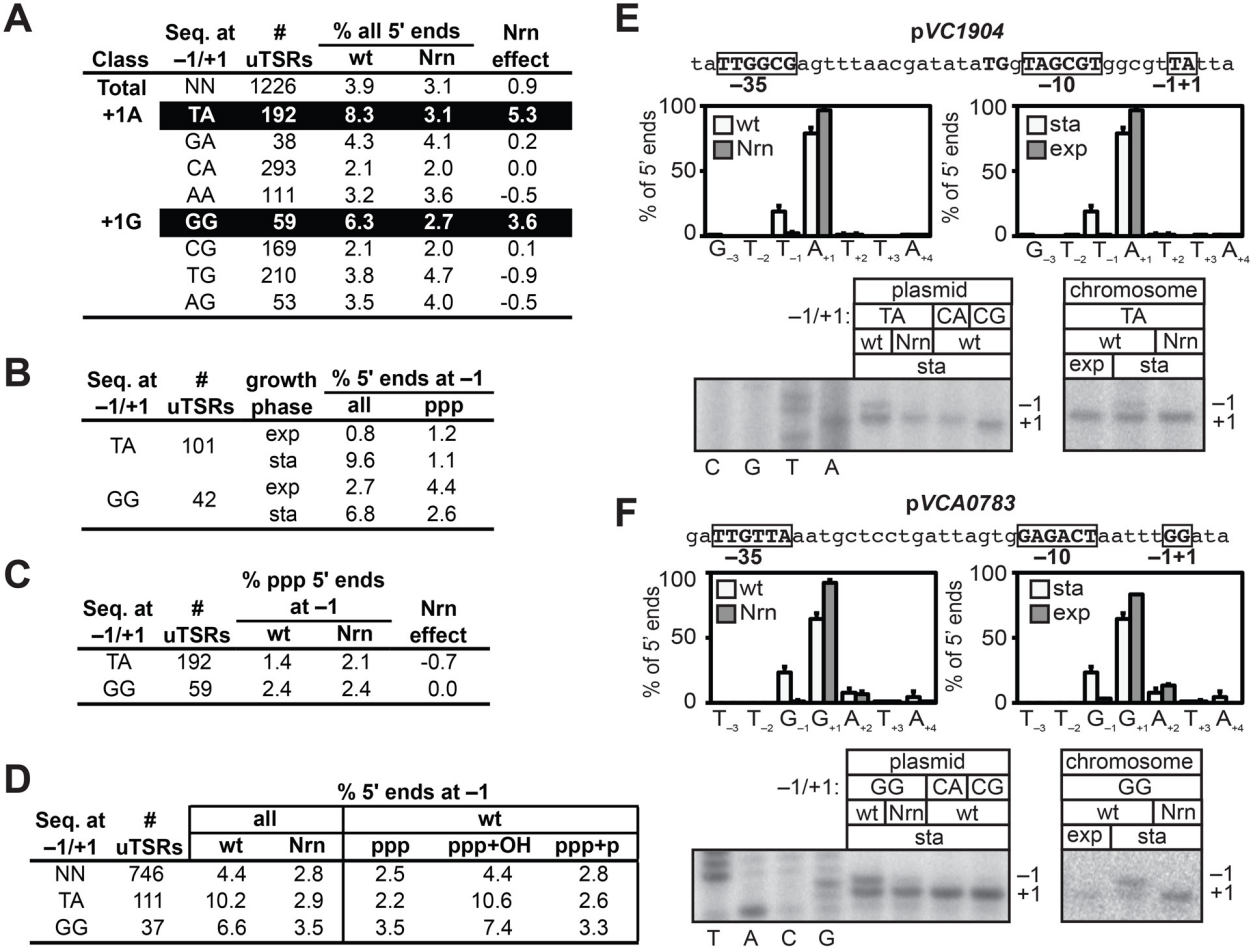
Detection of PDI in *V*. *cholera*. A-D. Analysis of PDI by 5′ RNA-seq. Percentage of transcripts emanating from position −1 of uTSRs (S2 and S3 and S4 Tables) with the indicated sequence at −1 and +1 in cells carrying wild-type concentrations of 2- to ~4-nt RNAs (wild-type) or cells in which the oligoRNase NrnB was ectopically expressed (Nrn). The Nrn effect reported in panels A and C represents the difference in these values. uTSRs with above average Nrn effect are highlighted in black. The total number of uTSRs used to calculate the percentages is indicated. Values in panels A and B are calculated from biological replicates listed in S2 Table, values in panel C is calculated from replicates listed in S3 Table and values in panel D are calculated from replicates listed in S4 Table. exp, exponential phase; sta, stationary phase; all, analysis of all 5′ ends; ppp, analysis of 5′ triphosphate ends; ppp+OH, analysis of 5′ triphosphate ends and 5′ hydroxyl ends; ppp+p, analysis of 5′ triphosphate ends and 5′ monophosphate ends. E. and F. Analysis of PDI at the promoters associated with *VC1904* (pVC1904) and *VCA0783* (p*VCA0783*). Top of each panel shows promoter sequence. Indicated are positions +1, −1 and the promoter −10 and −35 elements. Middle of each panel shows results of 5′ RNA-seq. Graphs on left show average distribution of all 5′ ends between positions −3 and +4 in cells carrying wild-type concentrations of 2- to ~4-nt RNAs (wt) or cells in which the oligoRNase NrnB was ectopically expressed (Nrn) as detected by 5′ RNA-seq during stationary phase. Graphs on the right show average distribution of all 5′ ends between positions −3 and +4 for p*VCA0783* in wild-type cells during stationary phase (sta) or exponential phase (exp). Bottom of each panel shows primer extension analysis of plasmid-borne promoter variants carrying the indicated sequences at positions −1/+1 (left) or primer extension analysis of chromosomally encoded promoters during exponential phase (exp) or stationary phase (sta).

To determine whether or not NrnB-sensitive transcripts in *V*. *cholerae* were preferentially generated from promoters carrying particular start site region sequences, we selected 1226 start site regions containing an unambiguous primary *de novo* start site (unambiguous Transcription Start site Regions, uTSRs). Specifically, uTSRs were identified as those where >75% of the total transcripts within the start site region emanate from position +1 in the analysis of triphosphate 5′ ends in cells containing wild-type concentrations of 2- to ~4-nt RNAs (S2 Table). As expected, given the bias for use of a purine NTP during *de novo* initiation observed in *E*. *coli* [19–21], we found that 1125 of the 1226 uTSRs (~92%) carried either an A or G at position +1. On average, in cells containing wild-type concentrations of 2- to ~4-nt RNAs, ~4% of the transcripts associated with these uTSRs emanated from position −1 in the analysis of all 5′ ends (Fig 3A). Furthermore, the proportion of transcripts initiating from position −1 was slightly reduced (to ~3%) in cells in which an NrnB was ectopically expressed (Fig 3A). We next separated the 1125 uTSRs where position +1 was an A or G into eight distinct classes on the basis of the identity of the bases at positions −1 and +1 and calculated the change in the percentage of transcripts emanating from position −1 upon ectopic expression of NrnB (Nrn effect) of each class (Fig 3A). Parsing the start site regions in this manner revealed that NrnB-sensitive transcripts initiating from position −1 are preferentially generated from uTSRs carrying T_−1_A_+1_ (an average Nrn effect of ~5% of the total transcripts for T_−1_A_+1_ start site regions versus an average Nrn effect of ~1% for all start sites regions) and G_−1_G_+1_ (an average Nrn effect of ~4% for G_−1_G_+1_ start site regions). Analysis of RNA transcripts isolated from cells carrying wild-type concentrations of 2- to ~4-nt RNAs during exponential phase indicates that the proportion of transcripts emanating from position −1 of T_−1_A_+1_ and G_−1_G_+1_ start site regions is significantly higher during stationary phase (9.6% for T_−1_A_+1_ and 6.8% for G_−1_G_+1_) compared with exponential phase (0.8% for T_−1_A_+1_ and 2.7% for G_−1_G_+1_) (Fig 3B and S3 Table), suggesting that the generation of NrnB-sensitive transcripts in *V*. *cholerae* is growth phase-dependent.

The NrnB-sensitive transcripts initiating from position −1 were detected in the analysis of all transcripts but were not detected in the analysis of transcripts carrying only 5′ triphosphate ends (Fig 3C). These findings indicated that the NrnB-sensitive transcripts identified in *V*. *cholerae* carried a 5′ hydroxyl or a 5′ monophosphate. We therefore repeated the 5′ RNA-seq analysis in a manner that would enable us to distinguish between transcripts carrying a 5′ hydroxyl and those carrying a 5′ monophosphate. Specifically, we analyzed transcripts carrying either a 5′ triphosphate or a 5′ monophosphate (ppp + p) or transcripts carrying either a 5′ triphosphate or a 5′ hydroxyl (ppp + OH). The NrnB-sensitive transcripts emanating from position −1 of T_−1_A_+1_ and G_−1_G_+1_ start site regions were only detected when 5′ hydroxyl transcripts were included in the analysis (Fig 3D and S4 Table). Thus, we conclude that the NrnB-sensitive transcripts emanating from position −1 of T_−1_A_+1_ and G_−1_G_+1_ start site regions carry a 5′ hydroxyl group.

To validate the 5′ RNA-seq analysis we performed primer extension to analyze transcripts generated from a promoter carrying a T_−1_A_+1_ start site region, p*VC1904* (Fig 3E, top), and a promoter carrying a G_−1_G_+1_ start site region, p*VCA0783* (Fig 3F, top). Consistent with the results of 5′ RNA-seq (Fig 3E and 3F, middle), growth phase-dependent NrnB-sensitive transcripts emanating from position −1 of p*VC1904* and position −1 of p*VCA0783* were detected by primer extension (Fig 3E and 3F, bottom). In addition, transcripts emanating from position −1 were not detected in the analysis of mutant derivatives of each promoter in which the wild-type sequence at positions −1 and +1 was changed to either C_−1_A_+1_ or C_−1_G_+1_ (Fig 3E and 3F, bottom). Thus, on the basis of both 5′ RNA-seq and primer extension analysis we conclude that growth phase-dependent PDI occurs in *V*. *cholerae*, that PDI in *V*. *cholerae* is preferentially apparent at promoters carrying T_−1_A_+1_ and G_−1_G_+1_ start site regions, and that PDI in *V*. *cholerae* leads to the generation of transcripts that carry a 5′ hydroxyl group.

### Reanalysis of PDI in *E*. *coli* reveals preferential targeting of both T_−1_A_+1_ and G_−1_G_+1_ start site regions

The preferential generation of oligoRNase-sensitive transcripts from T_−1_A_+1_ start site regions was observed in our prior analysis of PDI in *E*. *coli* [5], while the preferential generation of oligoRNase-sensitive transcripts from G_−1_G_+1_ start site regions was not (S5 and S6 Tables). Because our *V*. *cholerae* analysis used a 5′ RNA-seq protocol [16] that had been modified subsequent to our analysis of PDI in *E*. *coli*, we sought to establish whether or not the preferential targeting of G_−1_G_+1_ start site regions was a unique feature of PDI in *V*. *cholerae* or, alternatively, was not previously detected in *E*. *coli* due to technical limitations of our prior 5′ RNA-seq protocol [5]. We therefore reanalyzed the extent of PDI in *E*. *coli* during stationary phase using our modified 5′ RNA-seq protocol. As in our prior analysis [5], we identified several start site regions that contained NrnB-sensitive transcripts. Among these start site regions, we identified 50 where the proportion of transcripts emanating from template positions upstream of the primary start site (i.e. positions −3, −2, or −1) was reduced by >25% upon ectopic expression of NrnB (S7 Table).

**Fig 4:**
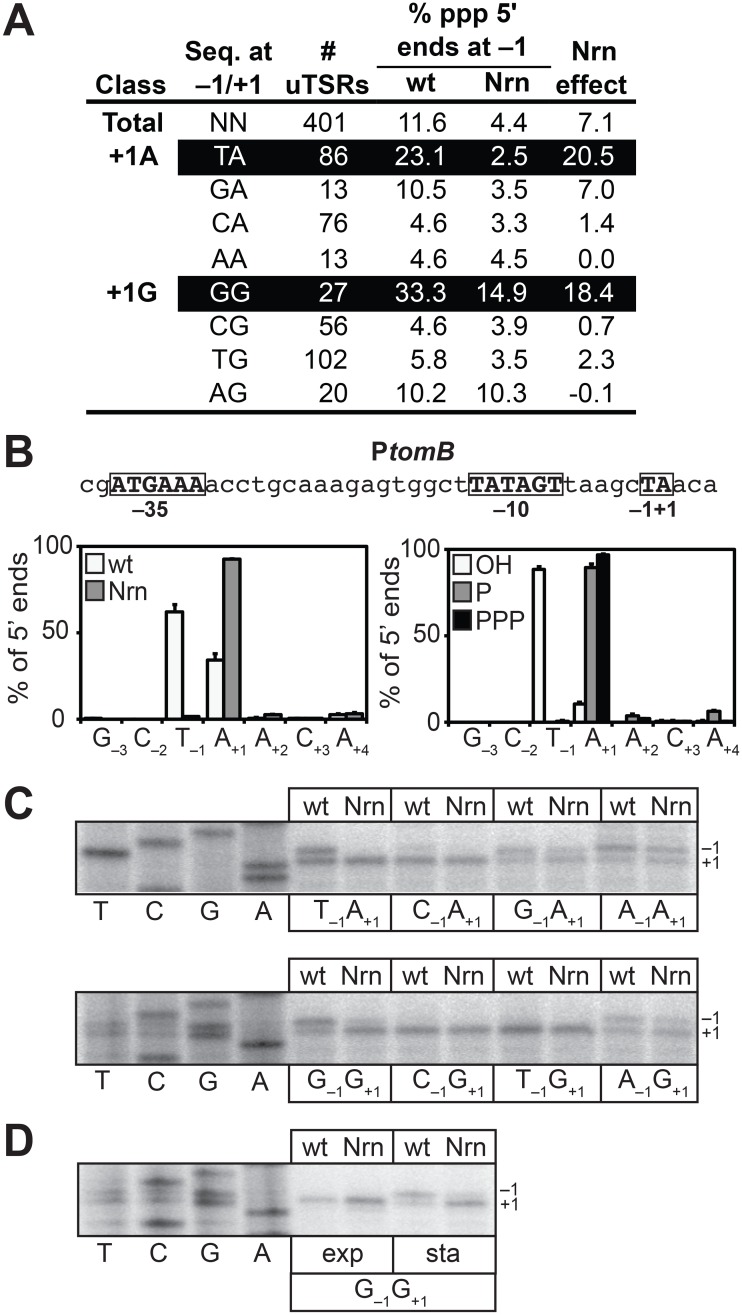
Detection of PDI in *E*. *coli*. A. Analysis of PDI by 5′ RNA-seq. Percentage of transcripts emanating from position −1 of uTSRs with the indicated sequence at −1 and +1 in cells carrying wild-type concentrations of 2- to ~4-nt RNAs (wild-type) or cells in which the oligoRNase NrnB was ectopically expressed (Nrn). The Nrn effect represents the difference in these values. uTSRs with above average Nrn effect are highlighted in black. The total number of uTSRs used to calculate the percentages is indicated. Values are calculated from biological replicates listed in S8 Table. Data is derived from the analysis of all 5′ ends during stationary phase. B. Analysis of PDI at the promoter associated with *tomB*, p*tomB*, by 5′ RNA-seq. Sequence of p*tomB* is shown. Indicated are positions +1, −1 and the promoter −10 and −35 elements. Graph on the left shows average distribution of 5′ ends between positions −3 and +4 for p*tomB* in cells carrying wild-type concentrations of 2- to ~4-nt RNAs or cells in which the oligoRNase NrnB was ectopically expressed (Nrn) as detected by 5′ RNA-seq analysis of all 5′ ends during stationary phase. Graph on the right shows the average distribution of 5′ ends between positions −3 and +4 for p*tomB* in cells carrying wild-type concentrations of 2- to ~4-nt RNAs as detected by 5′ RNA-seq analysis of hydroxyl 5′ ends (OH), monophosphate 5′ ends (P), or triphosphate 5′ ends (PPP) during stationary phase. C. Primer extension analysis of plasmid-borne p*tomB* variants carrying the indicated sequence at −1 and +1 in cells carrying wild-type concentrations of 2- to ~4-nt RNAs (wt) or cells in which the oligoRNase NrnB was ectopically expressed (Nrn). D. Primer extension analysis of the plasmid-borne p*tomB* variant carrying the sequence G_−1_G_+1_ during exponential phase (exp) or stationary phase (sta).

To facilitate a direct comparison of the results obtained in *E*. *coli* with those obtained in *V*. *cholerae* (Fig 3A), we identified 401 uTSRs (S8 Table). On average, in cells carrying wild-type concentrations of 2- to ~4-nt RNAs, ~11% of the transcripts associated with these 401 uTSRs emanated from position −1 in the analysis of all 5′ ends (Fig 4A). In contrast, the proportion of transcripts initiating from position −1 was reduced to ~4% in cells in which NrnB was ectopically expressed (Fig 4A). Thus, on average, ~7% of the total transcripts associated with a given uTSR are NrnB-sensitive in *E*. *coli*. Analysis of the 393 uTSRs where position +1 was an A or G revealed that, as in *V*. *cholerae*, NrnB-sensitive transcripts initiating from position −1 are preferentially generated from uTSRs carrying T_−1_A_+1_ (an average Nrn-effect of ~20% of the total transcripts for T_−1_A_+1_ start site regions versus an average Nrn-effect of ~7% for all start sites regions) and G_−1_G_+1_ (an average Nrn-effect of ~18% for G_−1_G_+1_ start site regions) (Fig 4A).

To validate the 5′ RNA-seq results obtained using our modified 5′ RNA-seq protocol we performed a systematic primer extension analysis of wild-type and mutant derivatives of the start site region associated with the *tomB* promoter (p*tomB*). We selected p*tomB* for this systematic analysis because it was among the promoters for which we detected NrnB-sensitive transcripts emanating from position −1 using both the prior version of the 5′ RNA-seq protocol [5] as well as the modified version (Fig 4B). The results show that the pattern of 5′ ends for *ptomB* derivatives observed using primer extension (Fig 4C) conforms to the rules inferred from the analysis of the 393 uTSRs using our modified 5′ RNA-seq protocol (Fig 4A). In particular, ~50% of the transcripts generated from the wild-type start site region (T_−1_A_+1_) or a mutant start site region carrying the sequence G_−1_G_+1_ emanate from position −1 and are significantly reduced upon ectopic expression of NrnB. In contrast, much lower levels of NrnB-sensitive transcripts emanating from position −1 are observed from derivatives with the sequence C_−1_A_+1_, C_−1_G_+1_, or T_−1_G_+1_. In addition, although a significant portion of transcripts generated from mutant derivatives carrying the sequence G_−1_A_+1_, A_−1_A_+1_, or A_−1_G_+1_ also emanate from position −1, only a small fraction of these transcripts appear NrnB-sensitive. Thus, on the basis of our 5′ RNA-seq and primer-extension analysis we conclude that PDI in *E*. *coli*, like in *V*. *cholerae*, is preferentially targeted to promoters carrying T_−1_A_+1_ and G_−1_G_+1_ start site regions. Because our prior 5′ RNA-seq protocol did not detect the preferential generation of oligoRNase-sensitive transcripts from G_−1_G_+1_ start site regions in *E*. *coli* [5] (S5 and S6 Tables), we further conclude that the modified 5′ RNA-seq protocol [16] is more sensitive and more accurate for the analysis of PDI *in vivo*. We suspect that the improved sensitivity of our modified 5′ RNA-seq protocol is a consequence of the use of a 5′ adaptor that carries six randomized bases at the 3′ end during Step 2 of the cDNA library construction (Fig 1B). Use of a 5′ adaptor carrying randomized bases at the 3′ end was included in our revised protocol to minimize the potential for sequence-dependent effects on ligation efficiency [22–25].

Our prior 5′ RNA-seq analysis in *E*. *coli* indicated that transcripts emanating from position −1 of T_−1_A_+1_ start site regions carry a 5′ hydroxyl [5]. To determine the phosphorylation state of the 5′ ends of transcripts emanating from position −1 of G_−1_G_+1_ start site regions we analyzed cDNA libraries generated from transcripts carrying only a 5′-hydroxyl or transcripts carrying only a 5′-monophosphate. As previously observed [5], transcripts emanating from position −1 of T_−1_A_+1_ start site regions were detected only in the analysis of transcripts carrying a 5′ hydroxyl (Fig 4B and S7 Table). Transcripts emanating from position −1 of G_−1_G_+1_ start site regions were also detected only in the analysis of transcripts carrying a 5′ hydroxyl (S1 Fig and S7 Table). We conclude that transcripts emanating from position −1 of G_−1_G_+1_ start site regions carry a 5′-hydroxyl, indicating that the primers used to generate these transcripts also carry a 5′-hydroxyl group in *E*. *coli*.

We have previously shown that oligoRNase-sensitive transcripts emanating from position −1 of T_−1_A_+1_ start site regions are not detected during exponential phase [5]. Primer extension analysis of a *tomB* promoter derivative carrying a G_−1_G_+1_ start site region indicates that transcripts emanating from position −1 are also not detected during exponential phase (Fig 4D). Thus, the appearance of transcripts produced by PDI from both G_−1_G_+1_ and T_−1_A_+1_ start site regions is growth phase-dependent in *E*. *coli*.

## Discussion

Here, we establish that PDI in *E*. *coli* serves as a regulatory mechanism important for biofilm formation (Fig 2). We further establish that PDI also occurs in *V*. *cholerae* (Fig 3 and S1, S2, S3, and S4 Tables) and that the pattern of PDI observed in *V*. *cholerae* is strikingly similar to that observed in *E*. *coli*. Specifically, PDI in both *E*. *coli* and *V*. *cholerae* is growth phase-dependent and preferentially targeted to promoters carrying start site regions with the sequence T_−1_A_+1_ or G_−1_G_+1_ (Figs 3 and 4). Our findings support an emerging model that PDI may be widespread in Gammaproteobacteria. To account for the preferential targeting of PDI to start site regions with the sequence T_−1_A_+1_ or G_−1_G_+1_, we propose that PDI in both *E*. *coli* and *V*. *cholerae* is a consequence of a growth phase-dependent process that leads to the preferential generation of the linear dinucleotides 5′-UA-3′ and 5′-GG-3′. It is well established that cyclic di-nucleotides can influence biofilm formation [26–30]. Our finding that PDI serves as a regulatory mechanism important for biofilm formation in *E*. *coli* (Fig 2) suggests that linear dinucleotides might also influence biofilm formation by serving as primers for transcription initiation.

### PDI as a mechanism for transcription initiation *in vivo*

The results described here, coupled with our prior analyses [5, 6], support an emerging model that PDI may be widespread in bacteria and that the extent of PDI relative to *de novo* initiation fluctuates as a function of growth state. During the growth conditions used in these studies we find that ~1% of transcripts generated from a given start site region in *V*. *cholerae* and ~7% of transcripts generated from a given start site region in *E*. *coli* are produced by PDI in late stationary phase (Figs 3A and 4A). However, the strict criteria we have used to measure PDI, which does not consider transcripts emanating from position +1 of a given start site region, and which considers only RNA transcripts emanating from start site regions that also produce products of *de novo* initiation, likely underestimates the full extent of PDI. In addition, the physiological triggers for PDI are unknown (see below). Thus, the particular growth conditions we have selected for our studies may not represent conditions where PDI is most prevalent. In this regard, over 50% of transcripts generated from a given start site region are produced by PDI in *P*. *aeruginosa* cells artificially depleted of the endogenous oligoRNase [6].

### A model for growth phase-dependent PDI

The PDI we have observed in *E*. *coli* and *V*. *cholerae* is detected in late stationary phase, is not detected in exponential phase, and is preferentially apparent at promoters carrying T_−1_A_+1_ and G_−1_G_+1_ start site regions. 5′ RNA-seq and primer extension analyses indicate that PDI from T_−1_A_+1_ and G_−1_G_+1_ start site regions produces transcripts emanating from position −1. Furthermore, the 5′ RNA-seq analysis indicates that transcripts emanating from position −1 of T_−1_A_+1_ and G_−1_G_+1_ start site regions begin with the sequences 5′-UA and 5′-GG, respectively. It follows that the primers used to generate these transcripts also begin with the sequence 5′-UA and 5′-GG, respectively. We therefore propose that the growth phase-dependent PDI observed in both *E*. *coli* and *V*. *cholerae* is the result of a process that leads to the generation of RNAs beginning with the sequence 5′-UA and 5′-GG at some stage during the transition from exponential phase to late stationary phase. Among all possible 2- to 4-nt RNAs beginning with 5′-UA and 5′-GG, only the linear dinucleotides 5′-UA-3′ and 5′-GG-3′ have the requisite template specificity to function as a primer during initiation at all T_−1_A_+1_ and G_−1_G_+1_ start site regions, respectively. Thus, the simplest model is that the linear dinucleotides 5′-UA-3′ and 5′-GG-3′ account for most, if not all, of the PDI observed from T_−1_A_+1_ and G_−1_G_+1_ start site regions.

Our 5′ RNA-seq analyses of sequence determinants that favor PDI considered only start site regions with an A or G at position +1. This was done to ensure that our conclusions were based upon the composite behavior of start site region sequences within the context of a significant number of distinct promoters. The finding that T_−1_A_+1_ and G_−1_G_+1_ start site regions are preferentially targeted among those that contain an A or G at +1 suggests that 2- to ~4-nt RNAs beginning with 5′-UA and 5′-GG are preferentially generated in cells relative to 2- to ~4-nt RNAs beginning with the sequence 5′-AA, 5′-CA, 5′-GA, 5′-AG, 5′-CG, or 5′-UG. However, our 5′ RNA-seq analysis of individual promoters in *V*. *cholerae* also detected oligoRNase-sensitive transcripts that began with the sequence 5′-UC (a promoter associated with VCA0982) (S1 Table). Furthermore, our 5′ RNA-seq analysis of individual promoters in *E*. *coli* detected oligoRNase-sensitive transcripts that began with the sequence 5′-CC (promoters associated with *ycjX*, *rplU*, and *iclR*) and 5′-UU (a promoter associated with *rpoS*) (S7 Table). Thus, in addition to RNAs beginning with the sequence 5′-UA and 5′-GG, other 2- to 4-nt RNA species may be present at concentrations sufficient to enable PDI in *V*. *cholerae* and *E*. *coli*.

We do not know the source of the oligoribonucleotides used as primers in *V*. *cholerae* and *E*. *coli*. In addition, we do not know whether or not the oligoribonucleotide primers are generated by a common mechanism in both organisms. One possibility is that oligoribonucleotides beginning with the sequence 5′-UA and 5′-GG are generated during RNA degradation. Another possibility is that the linear dinucleotides 5′-UA-3′ and 5′-GG-3′ are generated through a specific enzymatic process that awaits identification. In this regard, one intriguing speculation is that enzymes involved in the synthesis and/or breakdown of cyclic dinucleotides [27, 28, 30–34] may also have the ability to generate the linear dinucleotides 5′-UA-3′ and 5′-GG-3′, and this activity may become prevalent during stationary phase. Therefore, the cellular response to fluctuations in cyclic dinucleotide concentrations may also involve alterations in the extent of PDI. Furthermore, in principle, PDI may serve to control the intracellular concentrations of linear dinucleotides such as 5′-GG-3′ and 5′-UA-3′ by acting as a “sink” that removes such species from cells. Accordingly, PDI’s impact on cell physiology may extend beyond its effect on the composition of the transcriptome.

## Materials and Methods

### Strains

Experiments were performed using *E*. *coli* strain MG1655 and *V*. *cholerae* strain C6706*lacZ* [35]. *E*. *coli* strain MG1655 *ΔbhsA* (also called SG107) was constructed by using P1 transduction to move the *ΔbhsA*::*kan* locus from JW1098 *ΔbhsA*::*kan* [36] into MG1655.

### Plasmids

#### *V*. *cholerae* experiments

Plasmid pBAD-lacZ is used as an empty vector control for experiments performed in *V*. *cholerae* and has been described previously [37]. Plasmid pBADTOPO-NrnB-VSVG was generated by PCR amplification of *B*. *subtilis nrnB* from pNrnB-VSVG [5] using primers NrnBOVERFW (5′- TAAGAGGAATAATAAATGTATCATTTATATTCACATAACG-3′), NrnBOVERRV (5′-GTCGACCTATTTTCCTAATCTATTCATTTC-3′), and cloning of this PCR product into pBADTOPO according to the manufacturer’s instructions (Life Technologies). Plasmid pNT01 carries sequences extending from −100 to +15 of the promoter associated with VCA0783 (p*VCA0783*) fused to the tR' terminator cloned into the HindIII and SalI sites of plasmid pACYC184 (New England Biolabs). Plasmids pNT02 and pNT03 are identical to pNT01 with the exception that the start site region associated with p*VCA0783* carries the sequence C_−1_A_+1_ or C_−1_G_+1_, respectively. Plasmid pNT04 carries sequences extending from −100 to +15 of the promoter associated with VC1904 fused to the tR' terminator cloned into the HindIII and SalI sites of plasmid pACYC184. Plasmids pNT05 and pNT06 are identical to pNT04 with the exception that the start site region associated with p*VC1904* carries the sequence C_−1_A_+1_ or C_−1_G_+1_, respectively.

#### *E*. *coli* experiments

Plasmid pPSV38 is used as an empty vector control for experiments in *E*. *coli* and has been described previously [5]. Plasmid pNrnB-VSVG expresses the wild-type *nrnB* gene from *B*. *subtilis* strain PY73 fused to a VSV-G epitope tag under the control of an IPTG-inducible *lac*UV5 promoter flanked by two *lac* operators and has been described previously [5]. Plasmid pNrnB^DHH^-VSVG is the same as pNrnB-VSVG with the exception that the *nrnB* gene carries three amino acid substitutions, D86A, H87A and H88A [5]. pPSV38, pNrnB-VSVG and pNrnB^DHH^-VSVG confer resistance to gentamicin. Plasmid pBEN 504 has been described previously [5] and carries sequences extending from −100 to +15 of the promoter associated with *tomB* fused to the tR' terminator cloned into the HindIII and SalI sites of plasmid pACYC184. Seven derivatives of pBEN 504 were constructed that carry the indicated base-pairs at positions −1 and +1: pBEN 512 (C_−1_A_+1_), pBEN 556 (T_−1_G_+1_), pKS 415 (G_−1_G_+1_), pKS 416 (C_−1_G_+1_), pKS 417 (G_−1_A_+1_), pKS 421 (A_−1_A_+1_), and pKS 422 (A_−1_G_+1_). Plasmid pKS 385 carries sequences extending from −100 to +15 of the promoter associated with *ydiH* fused to the tR' terminator cloned into the HindIII and SalI sites of plasmid pACYC184. Two derivatives of pKS 385 were constructed that carry the indicated base pairs at positions −1 and +1: pKS 386 (C_−1_G_+1_) and pKS 387 (T_−1_G_+1_).

### Biofilm assays

Assays were performed using a procedure similar to that described in [18]. Plasmids pPSV38, pNrnB-VSVG or pNrnB^DHH^-VSVG were introduced into MG1655 or MG1655 *ΔbhsA* cells by transformation followed by plating on LB agar (LB-agar, Miller; EMD Millipore) containing gentamicin (10ug/mL), or gentamicin and kanamycin (50ug/mL). 5 ml overnights were grown in LB (Miller formulation: 10 g tryptone, 5 g yeast extract, and 10 g NaCl were mixed in deionized water, brought to pH 7 with 5 M NaOH and filter sterilized) containing 10 ug/ml gentamicin and 1 mM IPTG for 13.5 hours. Overnights were back-diluted 1:100 in 25 ml of LB broth containing 10 ug/ml gentamicin and 1 mM IPTG in a 125 ml DeLong flask (Bellco). Cultures were shaken at 220 RPM at 37°C to an OD_600_ of ~1.0 and 0.2 ml of the cell suspensions were placed in a 96-well microtiter plate (Greiner bio-one, cell culture treated U-bottom sterile microplate). After seeding, plates were sealed with breathable tape (World Wide Medical Products, bioexcell film for tissue culture) and incubated at 22.5°C for 32 hours. The growth medium was removed by aspiration and biofilms were washed twice with 0.15 ml of distilled water. The plate was inverted and then dried by incubating at 60°C for 2 hours. After the incubation, plates were cooled to 22.5°C and stained by addition of 0.2 ml of 0.1% crystal violet (w/v in water) for 12 minutes. The stain was removed by aspiration and the stained biofilms were washed once with 0.2 ml distilled water. The water was removed by aspiration and the plate was dried at 55°C for 5 minutes. The retained crystal violet stain was solubilized by addition of 220 μl of 33% acetic acid (v/v in water), incubation at 22.5°C for 1 hour, followed by vigorous mixing for 10 minutes. Afterwards, 100 μl aliquots of the released stain were transferred to a flat-bottom microplate and the absorbance was read at 570 nm using a microplate reader (Model 680, Biorad).

### Cell growth for 5′ RNA-seq

#### *V*. *cholerae* experiments

Plasmids pBAD-lacZ and pBADTOPO-NrnB-VSVG were introduced separately into C6706*lacZ* by transformation followed by plating on LB agar [1% (w/v) Tryptone, 0.5% (w/v) yeast extract, 1% (w/v) NaCl, 1.5% (w/v) agar] containing streptomycin (100 μg/ml) and carbenicillin (100 μg/ml). Experiments were performed in duplicate. Each transformant was used to inoculate 5 ml of LB broth [1% (w/v) Tryptone, 0.5% (w/v) yeast extract, 1% (w/v) NaCl] containing streptomycin (100 μg/ml) and carbenicillin (100 μg/ml) for ~18 hr at 37°C with shaking at 200 rpm. Overnight cultures were diluted to an optical density at 655 nm (OD_655_) of ~0.03 in 25 ml of LB broth containing streptomycin (100 μg/ml), carbenicillin (100 μg/ml) and L-arabinose [0.2% (w/v)] in a 125 ml flask (Kimax). Cultures were shaken at 220 RPM at 37°C and harvested after ~23 hr of growth (OD_655_ ~3.0). 10 ml of cell cultures were placed in 50 ml Falcon tubes (VWR) and centrifuged (9 min, 10,000 x g at 4°C) to collect cell pellets.

#### *E*. *coli* experiments

Plasmids pPSV38 and pNrnB-VSVG were each separately introduced into MG1655 cells by transformation followed by plating onto LB-agar (LB-agar, Miller; EMD Millipore) containing gentamycin (10 ug/mL). Experiments were performed using two independent transformants of each strain. Each transformant was used to inoculate 5 ml of LB (LB-broth, Miller; EMD Millipore) containing gentamycin (10 ug/mL) in an 18 mm x 150 mm glass culture tube. Tubes were placed in a tissue culture roller and cells were rotated for ~18 hr at 37°C. 250 ul of each overnight culture was added to 25 ml of LB containing gentamycin (10 ug/mL) and IPTG (1 mM) in a 125 ml DeLong flask (Bellco). Flasks were shaken at 220 RPM at 37°C and harvested after ~23 hr of growth (OD_600_ ~3.5). 5 ml of the cell suspensions were placed in 50 ml Oakridge tubes (Nalgene) and centrifuged (5 min, 10,000 x g at 4°C) to collect pellets. After removal of the supernatants, the cell pellets were stored at −80°C prior to RNA isolation.

### RNA isolation and purification

#### *V*. *cholerae* experiments

Cell pellets were resuspended in 2 ml of TRI Reagent solution (Molecular Research Center) and the suspension was split into two 1 ml aliquots that were placed into individual 2 mL microcentrifuge tubes. The suspensions were incubated at 60°C for 10 min, and centrifuged (10 min, 16,000 x g at 4°C). Each supernatant was transferred to a 2 ml tube, 200 μl of chloroform (Sigma) was added, the solution was mixed for 15 sec, incubated for 5 min at 25°C, and centrifuged (15 min, 16,000 x g at 4°C). 500 μl of each aqueous phase was added to a 2 ml tube containing 500 μl of 90% ethanol. Samples were mixed and transferred to RNeasy mini columns (Qiagen). RNA isolation was carried out according to the manufacturer’s instructions (Qiagen). RNA samples were eluted in 170 μl of nuclease-free water and treated with 20 units of Turbo DNase (Ambion) for 1 h at 37°C. 1 ml of TRI Reagent solution (Molecular Research Center) and 200 μl of chloroform (Sigma) were added, samples were mixed, incubated for 5 min at room temperature, and centrifuged (10 min, 16,000 x g at 4°C). 600 μl of the aqueous phase was transferred to a 2 ml tube containing 1.4 ml of 100% ethanol. Samples were incubated for ~12 h at −20°C, precipitated, washed twice with 1 ml of 75% ethanol, and resuspended in 50 μl of nuclease-free water.

#### *E*. *coli* experiments

Cell pellets were resuspended in 1 ml of TRI Reagent solution (Molecular Research Center), transferred to 1.7 ml low-binding microfuge tubes, incubated at 70°C for 10 min, and centrifuged (10 min, 21,000 x g at 4°C). The supernatant was transferred to a 1.7 ml tube, 200 μl of chloroform was added, the solution was mixed for 15 sec, incubated for 5 min at 25°C, and centrifuged (10 min, 21,000 at 4°C). 540 μl of the aqueous phase was transferred to a 1.7 ml tube, 1080 μl of 100% ethanol was added, samples were incubated at −80°C for a minimum of 2 hr, centrifuged (30 min, 21,000 at 4°C), washed with 1 ml of 75% ethanol, and resuspended in 25 μl of nuclease-free water. On-column DNase I (Qiagen) treatment was performed for 15 min at 25°C. RNA was eluted from the column into 30 μl nuclease-free water and stored at −80°C.

### 5′ RNA-seq: cDNA library construction

Prior to cDNA library construction the total RNA was passed through an RNeasy Mini Kit (Qiagen) to remove transcripts less than ~200 nt, and through a MICROBExpress Kit (Ambion) to remove some of the rRNAs. cDNA libraries suitable for sequencing on the Illumina HiSeq were constructed as described [16].

Ligation of the 5′ adaptor to the 5′ ends of input RNA (see Fig 1) requires the input RNA to carry a 5′ monophosphate. Thus, preparation of libraries derived from only those RNAs carrying a 5′ monophosphate requires no selective enzymatic treatments prior to ligation of the 5′ adaptor. In contrast, the exclusion of RNAs carrying a 5′ monophosphate in the analysis, the inclusion of 5′ triphosphate ends in the analysis, or the inclusion of 5′ hydroxyl ends in the analysis each requires selective enzymatic treatments to be performed prior to ligation of the 5′ adaptor. To prepare cDNA libraries derived from “all 5′ ends” rRNA-depleted RNA is treated with T4 Polynucleotide Kinase (NEB) to convert 5′ hydroxyl ends to 5′ monophosphate ends and RNA 5′-Polyphosphatase to convert 5′ triphosphate ends to 5′ monophosphate ends. To prepare cDNA libraries derived from 5′ hydroxyl ends and 5′ triphosphate ends rRNA-depleted RNA is treated with 5′-Terminator Exonuclease (Epicentre) to remove 5′ monophosphate ends, followed by T4 Polynucleotide Kinase and RNA 5`-Polyphosphatase. To prepare cDNA libraries derived from 5′ monophosphate ends and 5′ triphosphate ends rRNA-depleted RNA is treated RNA 5`-Polyphosphatase. To prepare cDNA libraries derived from 5′ triphosphate ends rRNA-depleted RNA is treated with 5′-Terminator Exonuclease followed by RNA 5`-Polyphosphatase. To prepare cDNA libraries derived from 5′ hydroxyl ends rRNA-depleted RNA is treated with 5′-Terminator Exonuclease followed by T4 Polynucleotide Kinase. Enzymatic treatments were done as described in [16].

### 5′ RNA-seq: Data analysis

The first six bases of each read was removed and Bowtie version 1.0.0 was used to align the raw reads to the *E*. *coli* reference genome (NC_000913.3) or the *V*. *cholerae* reference genome (NC_002505 and NC_002506). Alignment statistics are provided in S9 Table. Reads that aligned to a unique position in the genome with no mismatches were used to perform all analyses. This results in omission of non-unique sequences and excludes from analysis rRNA genes and some tRNA genes. Analysis of cDNA libraries derived from 5′ triphosphate transcripts isolated from cells carrying wild-type concentrations of 2- to ~4-nt RNAs were used to identify primary transcription start sites. In the case of the *V*. *cholerae* analysis, primary start sites were identified on the basis of the data obtained only from RNAs isolated during stationary phase. The read counts in biological replicates were combined and genomic coordinates that met the following two criteria were identified as primary start sites: First, the combined read count was at or above a threshold value of 50 reads. Second, the read count at the genomic coordinate represented a local maximum in an 11-bp window centered on the coordinate.

The read counts at each position within the start site regions (i.e. positions −3 to +4) associated with the primary start sites were used for subsequent analyses. The start site regions shown in S1 and S7 Tables represent those where the total read count was at or above a threshold value of 50 reads in each library derived from triphosphate 5′ ends or all 5′ ends and the proportion of 5′ ends at positions −3, −2, or −1 was reduced by >25% upon ectopic expression of NrnB. The “uTSRs” shown in S2, S4, S5 and S8 Tables meet the following two criteria. First, the proportion of transcripts initiating from position +1 is >75% in the analysis of triphosphate 5′ ends in cells carrying wild-type concentrations of 2- to ~4-nt RNAs. Second, the total read count was at or above a threshold value of 50 reads in each sample listed.

### 5′ RNA-seq: Data deposition

Raw reads have been deposited in the NIH/NCBI Sequence Read Archive under the study accession number SRP044667 (alias: PRJNA255655).

### Cell growth for primer extension

For the experiments shown in Fig 3, *V*. *cholerae* C6706*lacZ* cells carrying pBAD-lacZ or pBADTOPO-NrnB-VSVG were transformed with plasmid pNT01 (p*VCA0783*-G_−1_G_+1_), pNT02 (p*VCA0783*-C_−1_A_+1_), pNT03 (p*VCA0783*-C_−1_G_+1_), pNT04 (p*VC1904*-T_−1_A_+1_), pNT05 (p*VC1904*-C_−1_A_+1_), or pNT06 (p*VC1904*-C_−1_G_+1_). Cells were grown as described above with the exception that chloramphenicol (25 μg/ml) was also present in the growth media. RNAs for primer extension were isolated using Direct-zol RNA MiniPrep according to the manufacturer’s instructions (Zymo Research).

For the experiments shown in Fig 4 and S1 Fig, *E*. *coli* MG1655 cells carrying plasmids pPSV38 or pNrnB-VSVG were transformed with plasmids pBEN 504 (p*tomB*-T_−1_A_+1_), pBEN 512 (p*tomB*-C_−1_A_+1_), pBEN 556 (p*tomB*-T_−1_G_+1_), pKS 415 (p*tomB*-G_−1_G_+1_), pKS 416 (p*tomB*-C_−1_G_+1_), pKS 417 (p*tomB*-G_−1_A_+1_), pKS 421 (p*tomB*-A_−1_A_+1_), pKS 422 (p*tomB*-A_−1_G_+1_), pKS 385 (p*ydiH*-G_−1_G_+1_), pKS 386 (p*ydiH*-C_−1_G_+1_), or pKS 387 (p*ydiH*-T_−1_G_+1_). Cells were grown as described above with the exception that chloramphenicol (25 μg/ml) was also present in the growth media. RNAs were isolated from cells as described above.

### Primer extension

Primer extension analyses were performed using 10–20 μg of total RNA as previously described [6] with the following modifications. First, reverse transcription was performed at 55°C to minimize the addition of nontemplated nucleotides to the 3′ ends of the cDNA products [38]. Second, reactions performed using RNAs isolated from *V*. *cholerae* contained a final concentration of 1M Betaine (Affymetrix). Sequencing ladders were prepared using a Sequenase Version 2.0 DNA sequencing Kit (Affymetrix).

The following primers were used for the indicated loci:

Chromosomally-encoded p*VCA0783* (Fig 3): 5′-GGAAAGTAGTCGAGTCATGC-3′Chromosomally-encoded p*VC1904* (Fig 3): 5′-CACATCGGTTATACGGGCCGC-3′ Plasmid-encoded p*VCA0783* and p*VC1904* derivatives (Fig 3D and 3E): 5′-ATTTTGCAGACCTCTCTGCC-3′

Plasmid-encoded p*tomB* (Fig 4) and p*ydiH* (S1 Fig) derivatives: 5′-CCTCTCTGCCGGATCC-3′

## Supporting Information

S1 Fig Detection of PDI in *E*. *coli* at a promoter associated with *ydiH*, which carries a G_−1_G_+1_ start site region. A. Sequence of the *ydiH* promoter. Indicated are positions +1, −1 and the promoter −10 and −35 elements. B. Average distribution of 5′ ends between positions −3 and +4 for the *ydiH* promoter in cells carrying wild-type concentrations of 2- to ~4-nt RNAs (wt) or cells in which the oligoRNase NrnB was ectopically expressed (Nrn) as detected by 5′ RNA-seq analysis of all 5′ ends during stationary phase. Values are calculated from biological replicates listed in S7 Table. C. Average distribution of 5′ ends between positions −3 and +4 for the *ydiH* promoter in cells carrying wild-type concentrations of 2- to ~4-nt RNAs as detected by 5′ RNA-seq analysis of hydroxyl 5′ ends (OH), monophosphate 5′ ends (P), or triphosphate 5′ ends (PPP) during stationary phase. Values are calculated from biological replicates listed in S7 Table. D. Primer extension analysis of a plasmid-borne version of the *ydiH* promoter carrying a G_−1_G_+1_, C_−1_G_+1_, or T_−1_G_+1_ start site region during stationary phase. The results indicate oligoRNase-sensitive transcripts emanate from position −1 of only the wild-type *ydiH* promoter derivative that carries a G_−1_G_+1_ start site region.
(TIF)

S1 Table Analysis of PDI in *V*. *cholerae* by 5′ RNA-seq: Start site regions with >25% oligoRNase-sensitive transcripts during late stationary phase. Analysis of transcripts isolated during the late stationary phase of growth from *V*. *cholerae* cells harboring plasmid pBAD-lacZ (wt) or pBADTOPO-NrnB-VSVG (+Nrn). Shown for each start site region is: the promoter sequence extending from positions −40 to +4, the gene nearest to +1, the genome coordinate of position +1, the distance from +1 to the nearest gene (note that “–”indicates that +1 is upstream of the gene), and the percentage of transcripts sensitive to ectopic expression of the oligoribonuclease NrnB emanating from positions −1, −2 and −3 (% Nrn). The percentage of oligoRNase-sensitive transcripts was determined by calculating the difference between the percentage of reads at positions −1, −2 and −3 observed in wild-type cells from that observed in cells in which NrnB was ectopically expressed. Read counts derived from each position in the analysis of all 5' ends (5′ all) and 5' triphosphate ends (5′ ppp) are shown. Data obtained from *V*. *cholerae* set 1 (see S9 Table).
(XLSX)

S2 Table Analysis of PDI in *V*. *cholerae* by 5′ RNA-seq: Data for uTSRs during late stationary phase. Analysis of transcripts isolated during late stationary phase of growth from *V*. *cholerae* cells harboring plasmid pBAD-lacZ (wt) or pBADTOPO-NrnB-VSVG (+Nrn). Shown for each start site region is: the promoter sequence extending from positions −40 to +4, the sequence at positions −1 and +1, the genome coordinate of position +1, the gene nearest to +1, the distance of +1 to the nearest gene (note that “–”indicates that +1 is upstream of the gene), and the percentage of reads emanating from position +1 in the analysis of 5' triphosphate ends in wild-type cells. Read counts derived from the analysis of all 5' ends (5′ all) or 5' triphosphate ends (5′ ppp) are shown. Data obtained from *V*. *cholerae* set 1 (see S9 Table).
(XLSX)

S3 Table Analysis of PDI in *V*. *cholerae* by 5′ RNA-seq: Data for T_−1_A_+1_ and G_−1_G_+1_ start site regions. Analysis of transcripts isolated during late stationary phase or exponential phase from *V*. *cholerae* cells harboring plasmid pBAD-lacZ (wt) or pBADTOPO-NrnB-VSVG (+Nrn). Shown for each start site region is: the promoter sequence extending from positions −40 to +4, the sequence at positions −1 and +1, the genome coordinate of position +1, the gene nearest to +1, the distance of +1 to the nearest gene (note that “–”indicates that +1 is upstream of the gene), and the percentage of reads emanating from position +1 in the analysis of 5' triphosphate ends in wild-type cells during stationary phase. Read counts derived from the analysis of all 5' ends (5′ all) or 5' triphosphate ends (5′ ppp) are shown. The table contains data for 101 T_−1_A_+1_ uTSRs and 42 G_−1_G_+1_ uTSRs from S2 Table that also contained an above-threshold total read count (> 50) in the analysis of transcripts isolated during exponential phase. Data obtained from *V*. *cholerae* set 1 (see S9 Table).
(XLSX)

S4 Table Analysis of PDI in *V*. *cholerae* by 5′ RNA-seq: Data for uTSRs during late stationary phase. Analysis of transcripts isolated during late stationary phase of growth from *V*. *cholerae* cells harboring plasmid pBAD-lacZ (wt) or pBADTOPO-NrnB-VSVG (+Nrn). Shown for each start site region is: the promoter sequence extending from positions −40 to +4, the sequence at positions −1 and +1, the genome coordinate of position +1, the gene nearest to +1, the distance of +1 to the nearest gene (note that “–”indicates that +1 is upstream of the gene), and the percentage of reads emanating from position +1 in the analysis of 5' triphosphate ends in wild-type cells. Read counts derived from the analysis of all 5' ends (5′ all), 5' triphosphate ends (5′ ppp), 5' triphosphate ends and 5' hydroxyl ends (5′ ppp + OH), or 5' triphosphate ends and 5' monophosphate ends (5′ ppp +p) are shown. Data obtained from *V*. *cholerae* set 2 (see S9 Table).
(XLSX)

S5 Table Analysis of PDI in *E*. *coli* by 5′ RNA-seq: Data for uTSRs during late stationary phase using the prior 5′ RNA-seq protocol [5]. Analysis of transcripts isolated during the late stationary phase of growth from *E*. *coli* cells harboring plasmid pPSV38 (wt) or pNrnB-VSVG (+Nrn). Shown for each start site region is: the promoter sequence extending from positions −40 to +4, the sequence at positions −1 and +1, the genome coordinate of position +1, the gene nearest to +1, the distance of +1 to the nearest gene (note that “–”indicates that +1 is upstream of the gene), and the percentage of reads emanating from position +1 in the analysis of 5' triphosphate ends (% at +1). Read counts derived from the analysis of all 5' ends (5′ all) or 5' triphosphate ends (5′ ppp) are shown.
(XLSX)

S6 Table Analysis of PDI using the prior 5′ RNA-seq protocol [5]. Percentage of transcripts emanating from position −1 of uTSRs (S5 Table) with the indicated sequence at −1 and +1 in cells carrying wild-type concentrations of 2- to ~4-nt RNAs (wild-type) or cells in which the oligoribonuclease NrnB was ectopically expressed (Nrn). The Nrn effect represents the difference in these values. The total number of uTSRs used to calculate the percentages is indicated. Values are calculated from biological replicates listed in S5 Table. Data is derived from the analysis of all 5′ ends during stationary phase. uTSRs with above average Nrn effect are highlighted in black.
(XLSX)

S7 Table Analysis of PDI in *E*. *coli* by 5′ RNA-seq: Start site regions with >25% oligoRNase-sensitive transcripts during late stationary phase. Analysis of transcripts isolated during the late stationary phase of growth from *E*. *coli* cells harboring plasmid pPSV38 (wt) or pNrnB-VSVG (+Nrn). Shown for each start site region is: the promoter sequence extending from positions −40 to +4, the genome coordinate of position +1, the gene nearest to +1, the distance of +1 to the nearest gene (note that “–”indicates that +1 is upstream of the gene), and the percentage of transcripts sensitive to ectopic expression of the oligoribonuclease NrnB emanating from positions −1, −2 and −3 (% Nrn). The percentage of oligoRNase-sensitive transcripts was determined by calculating the difference between the percentage of reads at positions −1, −2 and −3 observed in wild-type cells from that observed in cells in which NrnB was ectopically expressed. Read counts derived from each position in the analysis of all 5' ends (5′ all), 5' triphosphate ends (5′ ppp), 5' hydroxyl ends (5′ OH), and 5' monophosphate ends (5′ p) are shown.
(XLSX)

S8 Table Analysis of PDI in *E*. *coli* by 5′ RNA-seq: Data for uTSRs during late stationary phase. Analysis of transcripts isolated during the late stationary phase of growth from *E*. *coli* cells harboring plasmid pPSV38 (wt) or pNrnB-VSVG (+Nrn). Shown for each start site region is: the promoter sequence extending from positions −40 to +4, the sequence at positions −1 and +1, the genome coordinate of position +1, the gene nearest to +1, the distance of +1 to the nearest gene (note that “–”indicates that +1 is upstream of the gene), and the percentage of reads emanating from position +1 in the analysis of 5' triphosphate ends (% at +1). Read counts derived from the analysis of all 5' ends (5′ all) or 5' triphosphate ends (5′ ppp) are shown.
(XLSX)

S9 Table Alignment statistics. Libraries VV362, VV363, VV366, VV367, VV370, VV371, VV372, and VV373 were generated using the prior iteration of the 5′ RNA-seq protocol (protocol 1) [5] while the other libraries listed were generated using the modified 5′ RNA-seq protocol (protocol 2) [16].
(XLSX)
