# Supplementary figures and images for: A Conserved Pattern of Primer-Dependent Transcription Initiation in Escherichia coli and Vibrio cholerae Revealed by 5′ RNA-seq
Source: PLoS Genet. 2015 Jul 1;11(7):e1005348. doi: 10.1371/journal.pgen.1005348 (PMC4488433; doi:10.1371/journal.pgen.1005348)

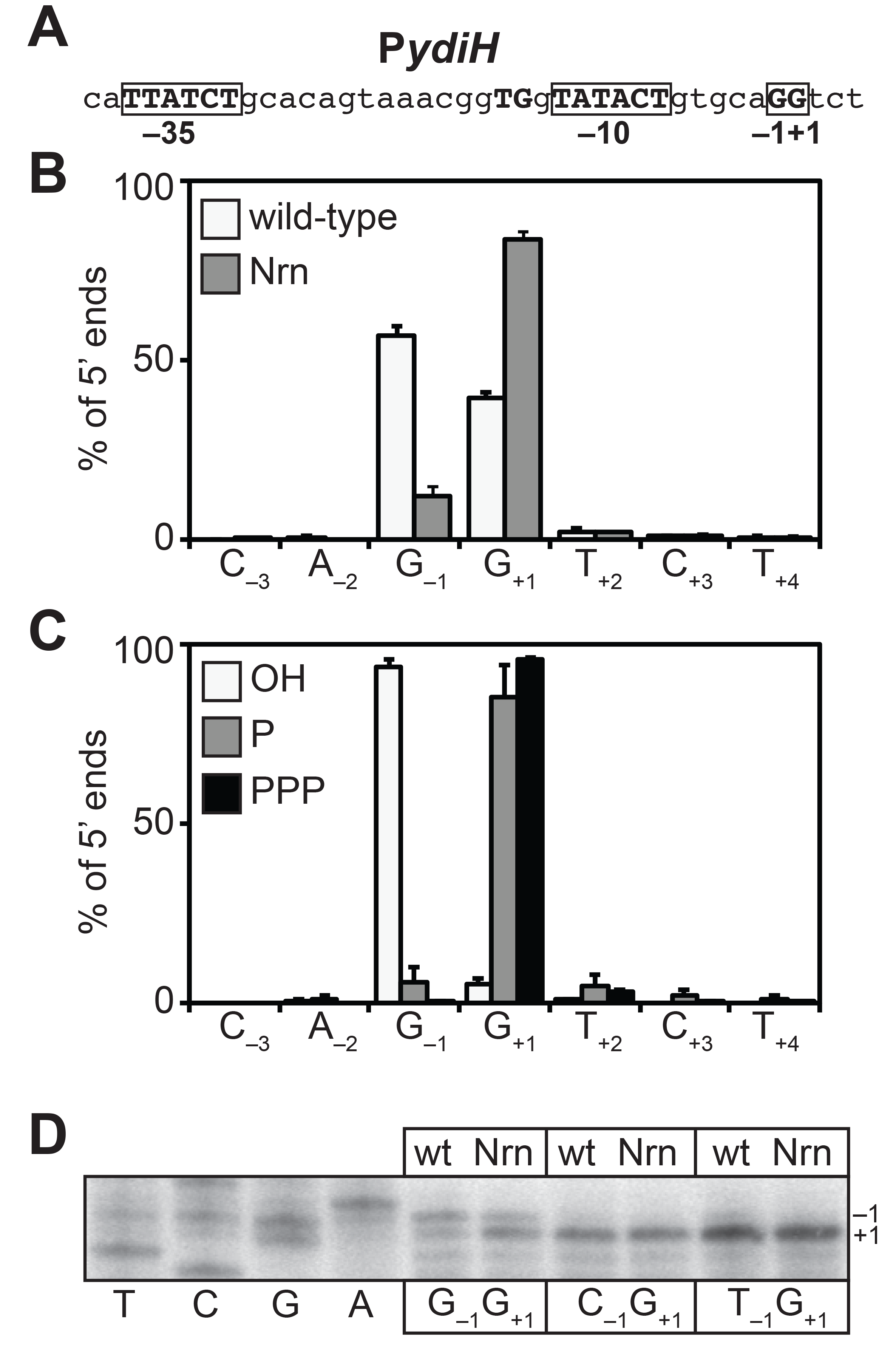

Supplement: S1 Fig — A. Sequence of the ydiH promoter. Indicated are positions +1, −1 and the promoter −10 and −35 elements. B. Average distribution of 5′ ends between positions −3 and +4 for the ydiH promoter in cells carrying wild-type concentrations of 2- to ~4-nt RNAs (wt) or cells in which the oligoRNase NrnB was ectopically expressed (Nrn) as detected by 5′ RNA-seq analysis of all 5′ ends during stationary phase. Values are calculated from biological replicates listed in S7 Table. C. Average distribution of 5′ ends between positions −3 and +4 for the ydiH promoter in cells carrying wild-type concentrations of 2- to ~4-nt RNAs as detected by 5′ RNA-seq analysis of hydroxyl 5′ ends (OH), monophosphate 5′ ends (P), or triphosphate 5′ ends (PPP) during stationary phase. Values are calculated from biological replicates listed in S7 Table. D. Primer extension analysis of a plasmid-borne version of the ydiH promoter carrying a G−1G+1, C−1G+1, or T−1G+1 start site region during stationary phase. The results indicate oligoRNase-sensitive transcripts emanate from position −1 of only the wild-type ydiH promoter derivative that carries a G−1G+1 start site region. (TIF) [file pgen.1005348.s001.tif]
